# A Diagnostic Tightrope: Carbimazole-Induced Microscopic Polyangiitis With Co-Existent Antiphospholipid Syndrome and Diffuse Alveolar Hemorrhage

**DOI:** 10.7759/cureus.105925

**Published:** 2026-03-26

**Authors:** P Thulasinadh, Alia Anzoom N, Maniyar Iqbal Anvar, Shashikanth Mallapur

**Affiliations:** 1 General Medicine, Ballari Medical College and Research Centre, Ballari, IND; 2 Nephrology, Ballari Medical College and Research Centre, Ballari, IND

**Keywords:** anca associated vasculitis, antiphospholipid syndrome, carbimazole, diffuse alveolar hemorrhage, microscopic polyangiitis, p-anca, plasmapheresis

## Abstract

Microscopic polyangiitis (MPA) and drug-induced vasculitis represent significant diagnostic challenges. Carbimazole-induced antineutrophil cytoplasmic antibodies (ANCA)-associated vasculitis is a rare, life-threatening complication of thionamide therapy. We describe a 29-year-old female patient on long-term carbimazole therapy for 15 years who presented with acute type-1 respiratory failure due to diffuse alveolar hemorrhage. Investigations confirmed myeloperoxidase (MPO)-ANCA-positive microscopic polyangiitis with co-existent definitive antiphospholipid syndrome and atypical "immune-complex-mediated" renal deposits. This case highlights the "triple-hit" of drug-induced vasculitis, thrombosis, and immune-complex deposition. The patient's rapid clinical stabilization following the initiation of plasmapheresis emphasizes the role of this intervention as a life-saving therapeutic bridge in complex vasculitis presentations.

## Introduction

Microscopic polyangiitis (MPA) is a systemic small-vessel vasculitis characterized by pauci-immune necrotizing glomerulonephritis and pulmonary involvement [1]. While primary MPA is idiopathic, drug-induced forms, particularly those triggered by thionamides like carbimazole, are increasingly recognized [2]. The diagnostic intersection of antineutrophil cytoplasmic antibodies (ANCA)-associated vasculitis (AAV) and antiphospholipid syndrome (APS) represents a high-stakes clinical scenario that complicates anticoagulation strategies [3]. Notably, the presence of atypical immune-complex deposits in certain patients suggests that drug-induced variants may possess a distinct immunopathological profile compared to idiopathic pauci-immune vasculitis [4].

## Case presentation

A 29-year-old female patient with hyperthyroidism, stable on carbimazole and propranolol for 15 years, presented with acute-onset hemoptysis and type 1 respiratory failure. Baseline investigations revealed significant anemia (hemoglobin 9.9 g/dL), leukocytosis (14,770 cells/mm^3^), and thrombocytopenia (94,000/mm^3^) with an elevated C-reactive protein (CRP) of 28.2 mg/L (Table 1). Her thyroid profile confirmed a hyperthyroid state consistent with her prolonged thionamide therapy, showing a TSH of 0.04 mIU/L and Free T4 of 30 ng/dL (Table 1). Suspecting drug-induced vasculitis, carbimazole was immediately discontinued.

**Table 1:** Baseline laboratory investigations WBC: white blood cell; ESR: erythrocyte sedimentation rate; CRP: C-reactive protein; PT: prothrombin time; INR: international normalized ratio; APTT: activated partial thromboplastin time; TSH: thyroid stimulating hormone; p-ANCA: perinuclear anti-neutrophil cytoplasmic antibody; MPO: myeloperoxidase; ANA: antinuclear antibody; IF: immunofluorescence; APLA: antiphospholipid antibodies; MPL: Mu-unit phospholipid units; CBNAAT: cartridge-based nucleic acid amplification test

Investigation	Patient Value	Reference Range
Hemoglobin	9.9 g/dL	12.0–15.5 g/dL
Total WBC Count	14,770 cells/mm^3^	4,500–11,000 cells/mm^3^
Differential Count	N94, L4	N: 40-75%, L: 20-45%
Platelet Count	94,000/mm^3^	150,000–450,000/mm^3^
ESR (1st hour)	15 mm	0–20 mm/hour
C-Reactive Protein (CRP)	28.2 mg/L	<5.0 mg/L
Prothrombin Time (PT)	12.5 seconds	11.0–13.5 seconds
INR	0.9	0.8–1.1
APTT	33.3 seconds	25.0–35.0 seconds
TSH	0.04 mIU/ml	0.5–5.0 mIU/ml
Free T3	9.98 pg/ml	2.3–4.2 pg/mL
Free T4	30 ng/dl	0.8–1.8 ng/dL
Urine Protein Creatinine Ratio	0.40	<0.20
P-ANCA (MPO)	2+	Negative
ANA (IF Method)	1:100 (2+)	<1:40 (Negative)
IgM Cardiolipin (APLA)	130.35 MPL	<20 MPL (Negative)
Sputum CBNAAT	Negative	Negative

**Figure 1:**
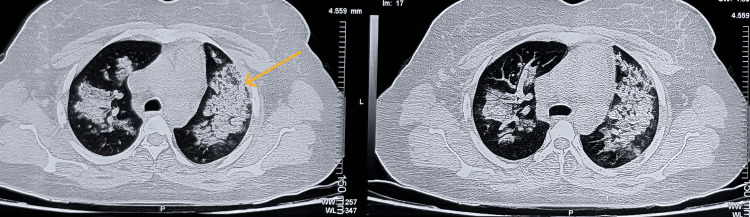
HRCT Thorax at admission showing diffuse alveolar hemorrhage. Extensive bilateral ground-glass opacities and alveolar infiltrates seen, consistent with acute diffuse alveolar hemorrhage. HRCT: high-resolution computed tomography

High-resolution computed tomography (HRCT) of the thorax on admission revealed extensive bilateral ground-glass opacities consistent with diffuse alveolar hemorrhage (DAH) (Figure 1). Concurrently, the immunological workup revealed 2+ p-ANCA (myeloperoxidase (MPO)) positivity. Reflecting this active multi-organ involvement, the patient’s Birmingham Vasculitis Activity Score (BVAS v3 [5,6]; used under license from Oxford University Innovation) was calculated at 14 (Table 2). Furthermore, the workup showed a significantly elevated IgM anticardiolipin antibody of 130.35 MPL. Evaluating this alongside her significant obstetric history using the 2023 American College of Rheumatology (ACR)/European Alliance of Associations for Rheumatology (EULAR) classification criteria for antiphospholipid syndrome (APS) [3], the patient scored 10 points (Table 3). This concurrent presumptive diagnosis of APS fundamentally guided our acute management strategy.

**Table 2:** Detailed BVAS v3 calculation The Birmingham Vasculitis Activity Score (BVAS) version 3 was utilized (under license from Oxford University Innovation) to quantify disease activity at the time of admission.

BVAS v3 Domain	Clinical/Laboratory Manifestation	Score
Respiratory	Hemoptysis and Diffuse Alveolar Hemorrhage (DAH)	6
Renal	Microscopic hematuria and Protein-creatinine ratio of 0.40	4
Systemic	Elevated CRP (28.2 mg/L) and malaise	4
Total Score		14

**Table 3:** 2023 ACR/EULAR classification criteria for APS applied The 2023 ACR/EULAR classification criteria for APS are adapted from Barbhaiya et al., 2023 [3] ACR: American College of Rheumatology; EULAR: European Alliance of Associations for Rheumatology; APS: antiphospholipid syndrome; IUD: Intra-uterine death; MPL: Mu-unit phospholipid units

Domain	Clinical / Laboratory Finding	Points
Obstetric	3 Late-term IUDs + 2 Early Abortions	5
Laboratory	High-titer IgM Anticardiolipin (>40 MPL)	5
Total Score	Definitive APS Classification	10

The patient was treated with pulse methylprednisolone (1 g intravenously once daily for three days), followed by the initiation of an intravenous cyclophosphamide induction protocol. Given the critical nature of this scenario, the patient also underwent five essential sessions of plasmapheresis, each involving a 1.0-fold plasma volume exchange utilizing 20% albumin and fresh frozen plasma as replacement fluids.

**Figure 2:**
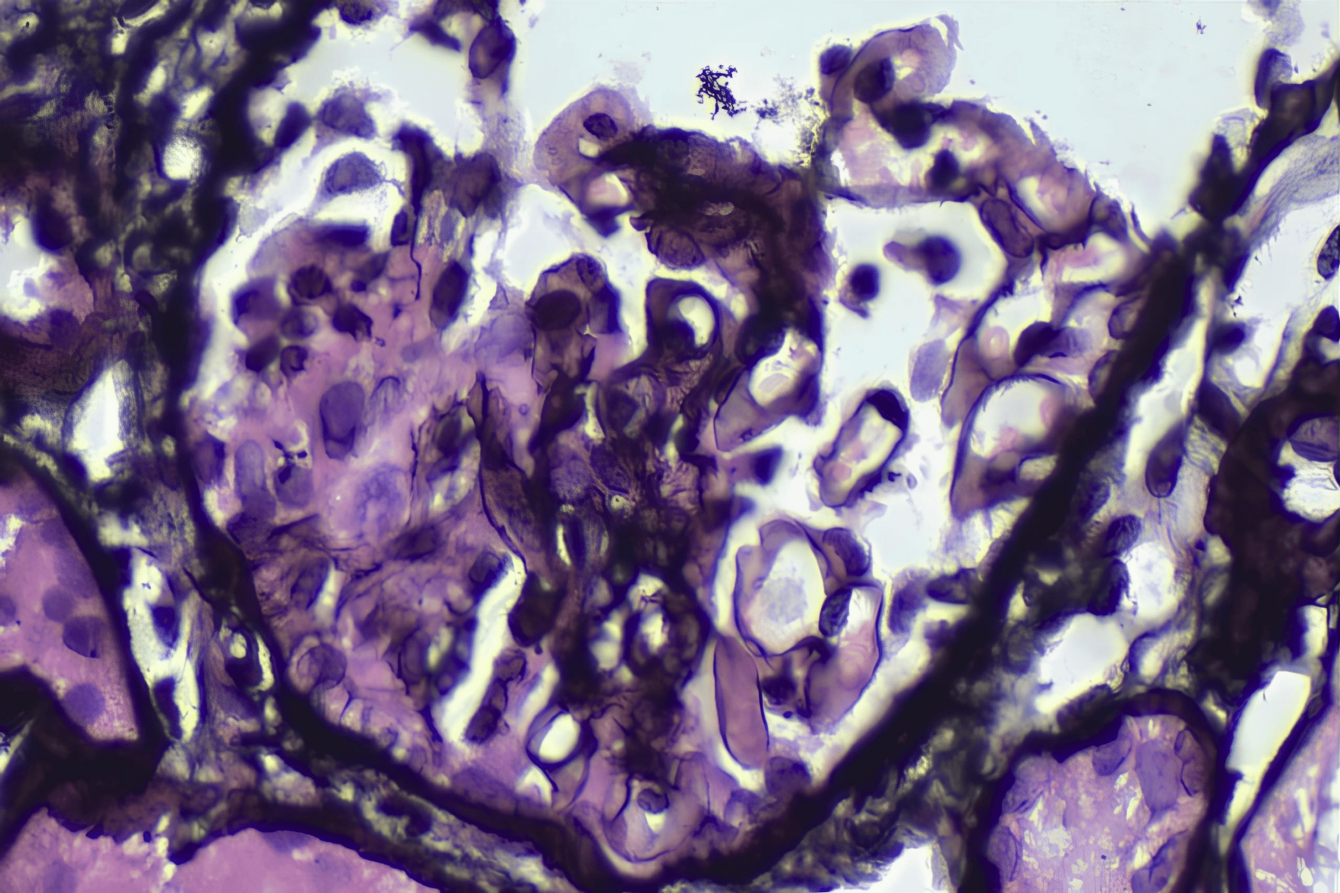
Light microscopy of renal biopsy (H&E or PAS stain). Light microscopy showing segmental tuft necrosis with disruption of basement membrane. PAS: periodic acid–Schiff

**Figure 3:**
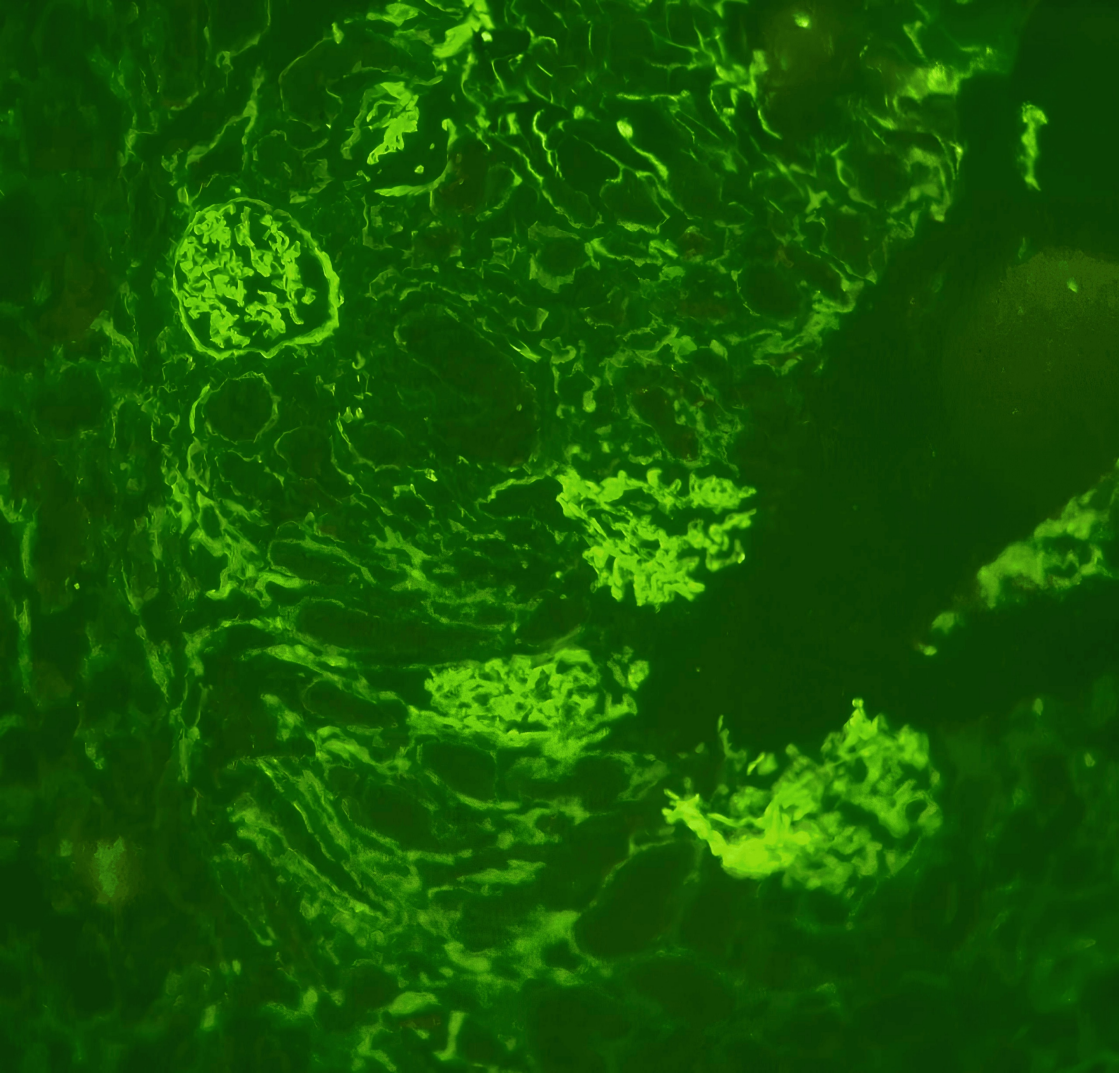
Immunofluorescence microscopy demonstrating moderate intensity granular immune deposits.

**Figure 4:**
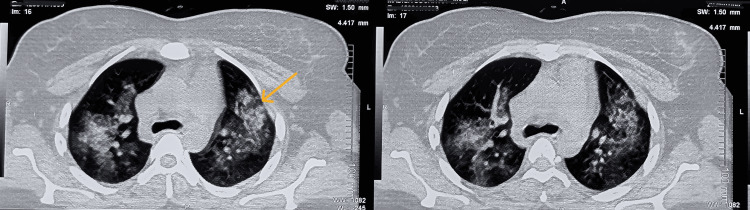
Post-treatment HRCT thorax showing resolution of hemorrhage. Near-complete resolution of DAH following pulse steroids and five cycles of plasmapheresis. DAH: diffuse alveolar hemorrhage

Renal evaluation showed microscopic hematuria and a protein-creatinine ratio of 0.40. A renal biopsy demonstrated segmental tuft necrosis with disruption of the basement membrane, highlighted on silver stain, in one glomerulus (Figure 2). There was no evidence of mesangial hypercellularity, and crescents were absent. The tubules showed patchy injury with luminal RBCs, while the interstitium exhibited mild diffuse lymphoplasmacytic cell infiltrate with minimal fibrosis. Notably, direct immunofluorescence revealed moderate intensity granular immune deposits (Figure 3), suggesting an "immune-complex-mediated" variant seen in approximately 25% of AAV cases [7]. Complement levels (C3 and C4) were tested and found to be within normal limits. Further characterization via immunoglobulins and electron microscopy (EM) was not performed due to institutional resource constraints. Following the initiation of pulse steroids and plasmapheresis, a repeat HRCT showed near-complete resolution of the prior opacities (Figure 4).

The patient completed the cumulative induction course of intravenous cyclophosphamide (500 mg every two weeks for a total of six doses over 14 weeks) and a tapering dose of oral prednisolone. Upon achieving clinical and radiological stability, a cautious rechallenge with carbimazole 10 mg was initiated to manage the underlying hyperthyroidism. The patient tolerated the rechallenge without a recurrence of vasculitis symptoms. Additionally, to address the APS component, the patient was started on aspirin (75 mg). Systemic anticoagulation was deferred since she had no prior history of thrombotic episodes and had already completed her obstetric goals. At the six-month follow-up, the patient remains in complete clinical and radiological remission with no recurrence of hemoptysis or renal dysfunction, and she maintains a euthyroid state with a TSH of 3.960 mIU/mL.

## Discussion

MPA and drug-induced AAV represent significant diagnostic challenges, particularly when triggered by thionamides like carbimazole. While thionamide-induced autoimmunity is well-documented, this case is unique due to the "triple-hit" of vasculitis, definitive APS, and atypical immune-complex deposition in a patient stable on therapy for 15 years. The pathogenesis of carbimazole-induced vasculitis likely involves the drug acting as a hapten that binds to MPO, altering its configuration and rendering it immunogenic. This triggers the production of MPO-ANCA (p-ANCA), which activates neutrophils to undergo "NETosis", the release of neutrophil extracellular traps, causing direct endothelial damage and the small-vessel capillaritis observed in diffuse alveolar hemorrhage (DAH). Unlike idiopathic MPA, drug-induced variants often present with higher ANCA titers and may involve co-existent immune-complex deposition, as seen in our patient’s renal biopsy [8].

The diagnostic intersection of AAV and APS represents a high-stakes clinical scenario that complicates standard management. Applying the 2023 ACR/EULAR classification criteria, the patient initially scored 10 points due to high-titer IgM anticardiolipin antibodies (>40 MPL) and a significant obstetric history. To satisfy the requirement for diagnostic rigor, the APLA profile was repeated 12 weeks post discharge. The persistence of IgM anticardiolipin positivity was crucial to distinguish definitive APS from a transient, drug-induced antibody elevation. Managing co-existent DAH and APS creates a profound therapeutic "tightrope", as APS necessitates anticoagulation while active DAH makes such therapy potentially fatal [9]. In this scenario, the initiation of five sessions of plasmapheresis, each involving a 1.0-fold plasma volume exchange with 20% albumin and fresh frozen plasma as replacement fluid, was essential. By providing mechanical clearance of both the pathogenic MPO-ANCA driving the hemorrhage and the pro-thrombotic factors, it acted as a life-saving bridge. This intervention led to rapid clinical stabilization and the conversion of p-ANCA to a negative status. A follow-up HRCT of the thorax demonstrated near-complete resolution of the diffuse alveolar hemorrhage (DAH) (Figure 4).

While the presence of ANA (2+) and APS antibodies could suggest systemic lupus erythematosus (SLE), the 15-year history of carbimazole use and the presence of normal serum complement levels (C3 and C4) and lack of full house effect in renal biopsy favor drug-induced MPA over a classic lupus-mediated flare. Following initial stabilization and completion of the intensive immunosuppressive regimen, a cautious rechallenge with carbimazole 10 mg was attempted. Unlike reported cases of thionamide-induced MPA where drug re-exposure often precipitates an immediate flare-up [10], the patient remained stable, achieving a euthyroid state (latest TSH: 3.960 mIU/mL). This successful reintroduction suggests that the intensive induction therapy provided a sufficient window of immunological suppression to safely reinitiate the necessary thionamide. This outcome underscores the importance of individualized, multidisciplinary management in balancing the control of life-threatening vasculitis with the need for long-term endocrine stability.

Following clinical resolution, the patient was transitioned to a maintenance regimen consisting of low-dose (10 mg) oral prednisolone, azathioprine, and to address the APS component, the patient was started on aspirin (75 mg). At the six-month follow-up, the patient remains in complete clinical and radiological remission with no recurrence of hemoptysis or renal dysfunction.

## Conclusions

This case highlights that thionamide-induced autoimmunity, specifically carbimazole-triggered MPA, remains a latent threat that can emerge even after 15 years of clinical stability. The presence of "atypical" granular immune deposits alongside MPO-ANCA suggests a hybrid immunopathological profile that deviates from the classic pauci-immune definition of idiopathic vasculitis, potentially representing a unique subset of drug-induced disease. The most critical management insight involves navigating the "therapeutic paradox" where the patient faces simultaneous life-threatening hemorrhage and high-risk thrombosis. In such instances, the rapid initiation of plasmapheresis is a decisive intervention; by mechanically extracting both pathogenic ANCA and pro-thrombotic anticardiolipin antibodies, it serves as an essential bridge that allows the pulmonary system to stabilize before long-term anticoagulation can be safely introduced. 

Furthermore, this clinical course challenges the standard dogma that the offending agent in drug-induced vasculitis must be permanently avoided. By implementing a robust induction regimen-consisting of six doses of intravenous cyclophosphamide over 14 weeks, we were able to achieve sufficient immunological suppression to allow for a successful, cautious rechallenge with carbimazole 10 mg. The patient’s subsequent transition to a stable, euthyroid state underscores that with aggressive, multidisciplinary oversight, it is possible to balance the control of systemic vasculitis with the necessity of managing the underlying endocrine disorder.
